# Addictions and risk behaviors in adolescence: a systematic review and qualitative analysis

**DOI:** 10.3389/fpsyg.2025.1646746

**Published:** 2025-09-25

**Authors:** María del Mar Simón Márquez, Silvia Fernández Gea, María del Mar Molero Jurado, Pablo Molina Moreno, María del Carmen Pérez-Fuentes

**Affiliations:** Department of Psychology, University of Almeria, Almería, Spain

**Keywords:** alcohol, tobacco, Cannabis, problematic internet use, adolescents, systematic review

## Abstract

**Introduction:**

Substance abuse and behavioral addictions among adolescents represent a growing public health concern, with significant implications for their physical, mental, and social development. This systematic review aimed to identify the most commonly used substances and prevalent addictive behaviors in this population.

**Methods:**

A comprehensive search was conducted across multiple scientific databases to select studies published between 2018 and 2023. Documents were analyzed qualitatively using the ATLAS.ti Web tool to identify patterns in the use of substances (alcohol, tobacco, cannabis, and cocaine) and behavioral addictions (problematic use of the internet, video games, gambling, smartphones, and social media).

**Results:**

After the systematic and manual selection process, a total of 41 research studies were obtained. Findings indicate that alcohol and tobacco are the most commonly used substances among European adolescents, with early onset linked to a higher risk of addiction. Excessive use of the internet and social media was also identified as a major concern, negatively impacting mental and social health. Video game and gambling addictions showed high prevalence rates, influenced by socioeconomic and family-related factors.

**Conclusion:**

These findings highlight that addictive behaviors in adolescents are multifaceted and influenced by biological, social, and environmental factors. There is a clear need for comprehensive prevention strategies that address both substance use and behavioral addictions to mitigate their impact on adolescent health.

## 1 Introduction

Adolescence is characterized as a critical period of life, in which a series of continuous biological, behavioral and social changes occur and foster human development (Bustamante et al., 2022). During the transition from childhood to adolescence, young people undergo significant changes in their brains. These changes occur in both the structure and function of the brain. They also take place in the social environment, particularly in the socio-emotional system due to puberty. This leads to an increased pursuit of rewards, especially in the company of peers (Steinberg, 2008; Viejo and Ortega-Ruiz, 2015). As a result, adolescence is a particularly vulnerable period for engaging in risky, problematic, and reckless behaviors, including cyber-aggression, violence within romantic relationships, and various addictive behaviors, which can have long-term consequences that negatively affect the transition and adjustment to adult life (Arrojo et al., 2024; Barragán et al., 2016; Molero et al., 2023).

The availability and consumption of addictive substances is a significant social issue, leading to serious health problems for young people and adversely affecting their environment (Pérez-Fuentes et al., 2018). In its Executive Report of the EDADES study (Ministerio de Sanidad, 2022), the Ministry of Health establishes that the onset of alcohol and tobacco consumption typically occurs between the ages of 16 and 17. The report also indicates that the consumption of alcohol, tobacco, cocaine, and cannabis is more frequent among males. Additionally, it highlights the prevalence of electronic cigarette and cannabis use among individuals aged 15 to 24 and identifies gambling, problem gambling, and gambling disorder as significant behavioral addictions.

Tobacco and alcohol consumption are the most common forms of substance abuse among European adolescents (Dudok and Piko, 2023). There is significant concern generated by traditional substance abuse and newer behavioral addictive habits. For this reason, numerous studies have been conducted to analyze this issue. For instance, Salamó et al. (2010) examine alcohol consumption patterns, social influence, and perceptions of danger. Another study carried out by Rial et al. (2020) explores the repercussions of early onset of consumption and seeks to identify related variables, such as substance use within the family environment or among peers. Currently, traditional cigarettes are not the only ones consumed. Electronic or vapor cigarettes are also widely used (Demissie et al., 2017).

According to the results of the HBSC Study (Ministerio de Sanidad, 2018), the percentage of individuals who report daily tobacco use increases with age, peaking among 17-18-year-olds. At this age, minimal differences in consumption are observed between males and females.

The HBSC Study (Ministerio de Sanidad, 2018) reveals that alcohol consumption increases with age, peaking among 17-18-year-olds. Gender differences become more pronounced with age, as males in this group report higher levels of alcohol consumption. Similarly, substance use, including drugs and alcohol, tends to rise with age, driven largely by curiosity, with men consuming more than women overall.

The HBSC Study further highlights that cannabis use is more prevalent among males than females, particularly within the 17-18-year-old demographic.

According to the ESPAD report, cannabis is the most commonly used illicit substance among European students. On average, 16% of respondents reported having used cannabis at least once, and 32% perceived it as easily accessible. Furthermore, 4% of students are considered at risk of severe dependency. This trend may be driven by the emergence of new cannabis variants and the increasing potency of its derivatives in recent years (Ministerio de Sanidad, 2020).

Other studies investigate the poly-consumption of substances. Barragán et al. (2016) examine the factors associated with alcohol, tobacco, and cannabis use among high school students; Peruga et al. (2022) analyze the use of electronic cigarettes and alcohol with this same population; and Calero-Plaza et al. (2020) explores adolescent poly-consumption of alcohol, cannabis, and marijuana, linking it to violent and delinquent behaviors.

On the other hand, behavioral addictions—addictions not linked to substances—are defined by impulsive and repetitive behaviors marked by a loss of control, a strong compulsion to engage in the behavior, and emotional distress. These behaviors persist despite serious consequences (De Sola et al., 2013).

Nowadays, technologies have become our inseparable companions and young people, in particular, are increasingly immersed in their use and embrace them with great enthusiasm. However, few are fully aware of the dangers they entail, such as cyberbullying, among other risks and challenges (Giménez-Gualdo et al., 2014; Molero et al., 2022, 2023).

Adolescents use Information and Communication Technologies (ICTs) more frequently and for longer hours, often without support from parental supervision. They use these tools in various settings, including educational centers, homes, and leisure contexts. Their purposes range from maintaining social relationships to completing academic tasks or entertaining themselves, either alone or with friends. This frequent use can lead to problematic behavior, especially among those aged 12 to 17 years (Gairín and Mercader, 2018).

Therefore, some studies analyze problematic internet use based on risk profiles, family characteristics, and behavioral and psychological factors (Arrivillaga et al., 2021). Other studies focus on the pathological use of video games (Hidalgo-Fuentes, 2023a; López-Fernández et al., 2020), gambling for money (Pereira, 2021), smartphone addiction (Salcedo and Lara, 2022; Hidalgo-Fuentes, 2023b; Díaz and Extremera, 2020), and social networks (Herrera-Peco et al., 2023; Muñoz and Ramírez, 2016).

Schools are essential for preventing substance use and supporting healthy decision-making, providing a space to identify at-risk youth early and offer them targeted interventions (European Monitoring Centre for Drugs and Drug Addiction, 2022). The significance of this systematic review lies in its broad and integrative approach, encompassing not only traditional substance abuse but also digital addictions linked to emerging technologies. By consolidating findings from diverse studies, this review aims to provide a comprehensive analysis of updated information regarding the most prevalent psychoactive substance use and other addictive behaviors among adolescents. This synthesis serves as a foundational resource for designing evidence-based prevention and intervention programs. These programs address behaviors that are pervasive in the lives of young people and pose serious risks to their physical and psychological health.

All this places adolescents in a highly vulnerable situation that can have severe consequences for their health (Morales-Rodríguez, 2022). The combination of substance and behavioral addictions in a single review responds to the need to comprehensively address the factors influencing addictive behaviors during adolescence. Both forms of addiction share neurobiological, psychological, and social characteristics, and their joint analysis allows for a broader understanding of the risks and vulnerability factors in this critical developmental stage. Therefore, the primary objective of this systematic review is to analyze updated information to describe the addictions most prevalent maladaptive behaviors among adolescents. By examining the most relevant data on this issue, this review aims to provide a foundation for prevention and intervention projects designed to reduce or eliminate these behaviors, which are pervasive in the daily lives of young people and detrimental to their physical and psychological health.

## 2 Materials and methods

### 2.1 Search process

The search was conducted in the ERIC, Psicodoc, PsycINFO, and Scopus databases, following the “PRISMA” methodology to ensure greater scientific rigor (Page et al., 2021). This search was performed in mid-April 2023, using Boolean operators in both Spanish (“conductas de riesgo” OR adicción OR ciberadicción) AND (adolescencia) and English (“risk behaviors” OR addiction OR cyber addiction) AND (adolescence). The initial results were filtered to include only scientific journal articles and were limited by date, covering the period from 2018 to April 2023, representing five full years and the month of April 2023. The PsycINFO database provided more precise filtering options in terms of subject, classification, and age group (see Table 1).

**Table 1:** Search results across different databases and applied filters.

Database	Terms language	Initial result	Article (scientific journal)	Date (2018–2023)	Subject (adolescent and risk factors)	Ranking (Substance abuse and addiction)	Age group (Adolescence)
ERIC	Spanish	0	0	0	0	0	0
	English	1,589	1,330	139	139	139	139
Psicodoc	Spanish	674	611	225	225	225	225
	English	704	656	107	107	107	107
PsycINFO	Spanish	20	20	13	13	13	13
	English	73,945	64,213	15,717	6,702	1,220	1,120
Scopus	Spanish	7	6	2	2	2	2
	English	2,088	1.628	458	458	458	458
The search yielded 2,064 results across the different databases.							

### 2.2 Inclusion and exclusion criteria

The results obtained were filtered manually by reading the titles and abstracts, followed by selection based on a series of inclusion and exclusion criteria. The inclusion criteria were: (a) the sample age should be between 12 and 17 years, inclusive, and (b) the study should describe adolescent addictive behaviors, providing relevant data.

The exclusion criteria were: (a) articles analyzing other problem behaviors not related to substance use and digital addictions, (b) documents where addictive behaviors are mentioned but the results do not provide significant contributions to understanding these behaviors, and (c) documents with samples younger than 12 years or older than 17 years.

### 2.3 Procedure for information analysis and selection process for publications

**Figure 1:**
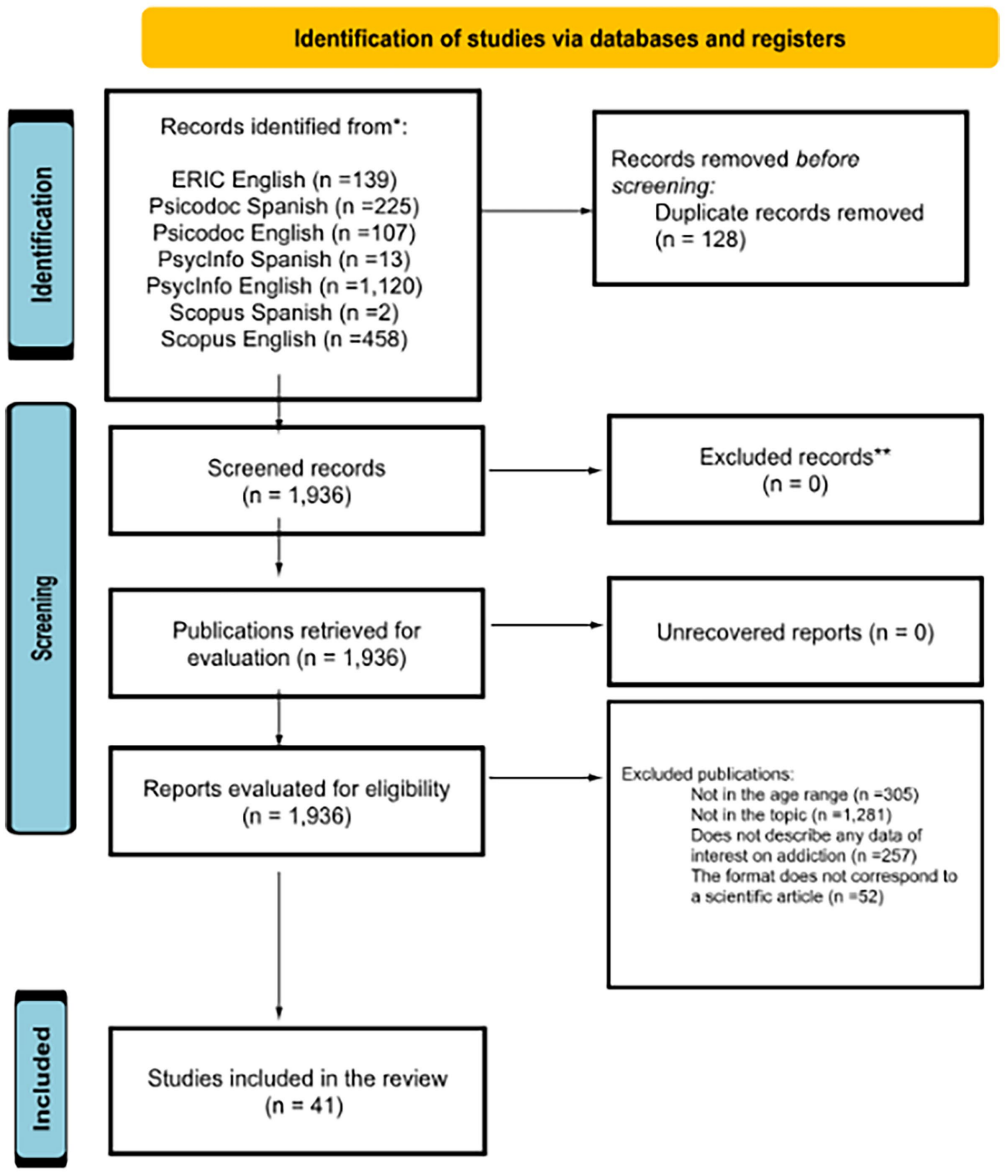
Search equation and flowchart of the document selection process.

Below is the flow diagram that summarizes the stages of the search and study selection process. This scheme, based on the PRISMA guidelines (Page et al., 2021), clearly illustrates the number of records identified, screened, excluded, and ultimately included in the review (Figure 1).

### 2.4 Coding data

After obtaining 2,064 documents from various databases, the distribution was as follows: ERIC with search in Spanish (0), ERIC with search in English (139), Psicodoc with search in Spanish (225), Psicodoc with search in English (107), PsycINFO with search in Spanish (13), PsycINFO with search in English (1,120), Scopus with search in Spanish (2), and Scopus with search in English (458).

Duplicate articles, totaling 128, were subtracted from these results: Psicodoc with search in Spanish (1), Psicodoc with search in English (70), PsycINFO with search in Spanish (6), PsycINFO with search in English (12), Scopus with search in Spanish (1), and Scopus with search in English (38).

After eliminating duplicate articles, a total of 1,936 documents were obtained. Titles and abstracts were then reviewed, and exclusion criteria were applied: (a) the document addresses a different subject matter than the one addressed here, (b) the age range of the participants in the studies is outside the range established for this systematic review, and (c) the document relates addiction to another subject but does not provide the necessary results regarding the subject matter of this systematic review. The following articles were eliminated: ERIC with search in English (a = 126, b = 6, c = 1), Psicodoc with search in Spanish (a = 103, b = 64, c = 15), Psicodoc with search in English (a = 23, b = 4, c = 8), PsycInfo with search in Spanish (a = 6, b = 1, c = 0), PsycInfo with search in English (a = 762, b = 159 and c = 152), Scopus with search in Spanish (a = 0, b = 1, c = 0), and finally, Scopus with search in English (a = 261, b = 61, c = 81). Additionally, 52 articles were excluded for not adhering to the scientific format: ERIC with search in English (5), Psicodoc with search in Spanish (20), Psicodoc with search in English (1), PsycInfo with search in English (19), and lastly, Scopus with search in English (7).

A total of 51 documents were obtained, which were manually filtered after reading the entire document to eliminate those that did not meet the inclusion and exclusion criteria. Consequently, 10 documents were excluded because, although their abstracts presented a mean age that matched the criteria established in this systematic review, the methodology section, specifically the participant section, revealed that the age range did not meet this criterion. These exclusions were from the following databases: ERIC with search in English (1), Psicodoc with search in Spanish (2), PsycInfo with search in English (6), and Scopus with search in English (6).

A total of 41 documents were obtained from the following databases: ERIC with search in English (1), Psicodoc with search in Spanish (20), Psicodoc with search in English (1), PsycInfo with search in English (10), and Scopus with search in English (9).

## 3 Results

After the systematic and manual selection process, a total of 41 quantitative research studies were obtained. These studies describe the most common addictive patterns among adolescents, which include smartphones, social networks, internet, gambling, video games, tobacco, alcohol, cannabis, and cocaine (see Table 2).

**Table 2:** Scientific studies on the most common addictions among adolescents.

Author(s)	Country	Sample size	Age	Abuse of	QualSyst score (%)
Lee et al. (2018)	South Korea	490	12–15 years old (*M* = 14,89)	Smartphone	95.45%
Dos Santos et al. (2018)	Brazil	153	14–17 years old	Alcohol	90%
Rodrigues et al. (2018)	Brazil	122	13–16 years old (*M* = 14,84)	Alcohol	90.91%
González-Ramos et al. (2018)	Spain	2,205	14–16 years old (*M* = 14,79)	Alcohol	86.36%
Díaz et al. (2018)	Spain	238	13–16 years old (*M* = 14,90)	Alcohol, tobacco and cannabis.	100%
Peris et al. (2018)	Spain	2,417	12–17 years old	Social networks and internet	100%
Martínez-Fernández et al. (2018)	Spain	126	12–13 years old (*M* = 12,17)	Alcohol, tobacco and cannabis	86.36%
Machado et al. (2018)	Brazil	91	12–16 years old (*M* = 12,80)	Internet	86.36%
Matalí et al. (2018)	Spain	228	*M* = 15.7 years	Cannabis	100%
Caselles et al. (2018)	Spain	2,716	13–17 years old (*M* = 15,12)	Gambling	90.91%
Wicki et al. (2018)	Switzerland	1,333	14–15 years (*M* = 14,60)	Alcohol	95.45%
Calero et al. (2019)	Argentina	399	(*M* = 15,14)	Alcohol	90.91%
Bekir and Çelik (2019)	Turkey	214	14–17 years old	Video games	90.91%
Camelo et al. (2019)	Colombia	204	12–17 years old (*M* = 14,50)	Alcohol, tobacco and cannabis	90.91%
Cimadevilla et al. (2019)	Spain	357	13–17 years old (*M* = 14,92)	Internet and Smartphone	90.91%
Villanueva et al. (2019)	Spain	284	14–17 years old (*M* = 15,23)	Alcohol, tobacco and cannabis	90.91%
Grande-Gosende et al. (2019)	Spain	1,810	14–17 years old (*M* =15,24)	Video games and gambling	90.91%
Silveira et al. (2019)	USA	6,127	15–17 years old.	Alcohol, tobacco and cannabis	90.91%
Jones and McCance-Katz (2019)	USA	27,900	12–17 years old	Alcohol, tobacco and cannabis	100%
Martínez-Loredo et al. (2019)	Spain	1,644	(*M* =15,22)	Gambling and alcohol	100%
Clements-Nolle et al. (2019)	USA	1,079	12–17 years old (*M* = 15,30)	Alcohol	100%
Strong et al. (2019)	USA	13, 651	12–17 years old	Tobacco	95.45%
Rial et al. (2020)	Spain	3,419	(*M* = 14,57)	Alcohol	95.45%
Buja et al. (2020)	Italy	15,833	14–17 years old	Gambling	95.45%
Zammit et al. (2018)	Tunisia	4,272	(*M* = 13,30)	Tobacco	100%
Lee et al. (2020)	South Korea	555	14–15 years	Internet and smartphone	100%
Dou et al. (2020)	China	333	15–16 years (*M* = 15,37)	Smartphone	100%
López-Fernández et al. (2021)	Spain	776	12–17 years old (*M* = 14,29)	Video games	100%
González-Roz et al. (2021)	Spain	107	12-to 17 years old (*M* = 15,46)	Alcohol, tobacco and cannabis	86.36%
Wartberg et al. (2021)	Germany	515	(*M* = 16,60)	Alcohol	100%
Tackett et al. (2021)	USA	507	13–17 years old (*M* = 15,90)	Tobacco	100%
Kaya and Dalgiç (2021)	Turkey	367	12–17 years old (*M* = 14,38)	Internet	90.91%
Macur and Pontes (2021)	Slovenia	1,071	12–16 years old (*M* = 13,44)	Video games	95.45%
Isorna et al. (2022)	Spain	3,108	12–17 years old (*M* = 14,44)	Internet, gambling, tobacco, alcohol, cocaine and cannabis	100%
Pastor et al. (2022)	Spain	524	13.57 years (*M* = 13,57)	Smartphone and internet	86.36%
Hernández et al. (2022)	Mexico	32	12–16 years old (*M* =13,19)	Alcohol	79.17%
Hing et al. (2022)	Australia	841	12–17 years old (*M* =14,70)	Gambling	90.91%
Hahlbeck and Vito (2022)	USA	2,043	12–17 years old	Cannabis	90.91%
Warburton et al. (2022)	Australia	866	12–17 years old (*M* = 14,42)	Video games	100%
Ricci et al. (2023)	Italy	2	15–16 years (*M* = 15,50)	Video games	92.86%
Richard et al. (2023)	USA	5,997	12–17 years old (*M* = 14,70)	Gambling	90.91%

### 3.1 Quality assessment of studies

The methodological quality of the included studies was assessed using the “QualSyst” scale (Kmet et al., 2004), which provides a standardized framework for appraising both quantitative and qualitative research based on criteria related to study design, sampling, analysis, and reporting (14 items for quantitative studies and 10 for qualitative studies). Each item is rated on a scale from 0 to 2, with the denominator adjusted when a criterion is not applicable, yielding a final score expressed as a percentage. According to this metric, values above 75% are classified as high quality, those between 55–75% as moderate quality, and those below 55% as low quality. In the present study, scores ranged from 79.19 to 100%, which overall reflects a high methodological quality (see Table 2).

### 3.2 Country where the research was conducted and types of addictions or problematic behaviors studied

A total of 41 studies conducted across 15 countries were included in this review. The largest proportion of studies originated from Spain (*n* = 15), followed by the United States (*n* = 7) and Brazil (*n* = 3). Other countries represented were South Korea (*n* = 2), Italy (*n* = 2), Turkey (*n* = 2), Australia (*n* = 2), and single studies from Argentina, Switzerland, Colombia, China, Tunisia, Germany, Slovenia, and Mexico.

Regarding the types of addictions, categories were not mutually exclusive, as several studies examined multiple behaviors or substance uses. The most frequently investigated was alcohol use (*n* = 18), followed by tobacco use (*n* = 11), cannabis use (*n* = 10), problematic internet use (*n* = 8), gambling (*n* = 7), video gaming (*n* = 6), and problematic smartphone use (*n* = 5).

### 3.3 Sample size, age of sample, and study design

Regarding the size of the study samples, there is notable variety, ranging from general studies with 27,900 participants to more personal studies with just 2 participants. Specifically, 4.88% of the articles have a sample size of over 15,000 users, 2.44% have between 10,000 and 14,000 users, 4.88% have between 10,000 and 15,000 users, 26.83% have between 5,000 and 10,000 users, 17.07% have between 500 and 1,000 users, 36.58% have between 100 and 500 users, and 7.32% have fewer than 100 users.

The age range for the established sample was 12 to 17 years, as selected for the systematic review. However, about a third of the selected studies have fully addressed this stage of adolescence. The most frequently cited age in the articles was 15 years, while the least cited was 12 years. This is an important aspect to consider, since addictions often begin at early ages.

### 3.4 Analysis of results

The review of evidence provides a comprehensive and multidimensional perspective on the factors associated with the use of substances (alcohol, tobacco, cannabis, cocaine) and problematic technology use (mobile phones, internet, video games, social media), as well as the phenomenon of polyconsumption among adolescents. These behaviors, rooted in a complex interaction of motivational, familial, social, and psychological variables, are characterized by their heterogeneity and high comorbidity.

Regarding alcohol consumption, the primary motivations include the pursuit of fun, social disinhibition, avoidance of emotional distress, and peer pressure. These motivations exhibit gender differences, with adolescent girls often justifying their consumption for social acceptance, while boys associate it with reinforcing ideals of masculinity (Dos Santos et al., 2018). Family environment is also a critical factor; according to (Rodrigues et al., 2018), 40% of adolescents reported a family history of excessive alcohol use, with first exposure commonly occurring at home. Additionally, peer pressure from high-risk groups and a lack of parental control exacerbate consumption (González-Ramos et al., 2018). Older adolescents with greater purchasing power are at significantly higher risk (Díaz et al., 2018). The consequences include tolerance to negative effects such as hangovers or shame, while academic and social implications receive less acknowledgment (Wicki et al., 2018). Early initiation (before age 14) is associated with a higher risk of problematic consumption and antisocial behaviors (Villanueva et al., 2019; Rial et al., 2020). Protective factors include positive family relationships, community involvement, and strategies focusing on self-efficacy and peer support (Clements-Nolle et al., 2019; Hernández et al., 2022).

Tobacco consumption is similarly influenced by economic and social factors, such as financial accessibility and sensation-seeking, particularly among men (Díaz et al., 2018; Martínez-Fernández et al., 2018). Early initiation increases the risk of antisocial behavior and cognitive deterioration (Villanueva et al., 2019). Additionally, electronic cigarettes, often mistakenly perceived as less harmful, promote dual use with traditional cigarettes, significantly heightening dependence (Strong et al., 2019; Tackett et al., 2021).

Similarly, cannabis use often begins at an early age, with an average initiation age of 12.9 years, and is associated with risks such as dependency (67.1% in regular users) and progression to stronger substances like opioids and methamphetamines (Jones and McCance-Katz, 2019; Matalí et al., 2018). Impulsivity and frequent alcohol consumption further elevate this risk (González-Roz et al., 2021). Furthermore, cannabis consumption is linked to the development of psychopathologies (Díaz et al., 2018).

Problematic mobile phone use negatively impacts psychological health, reducing self-esteem, sleep quality, and social relationships. It is associated with aggressive behaviors, low self-control, and increased risk-taking, with only 25% of adolescents managing to self-regulate their usage (Pastor et al., 2022). Girls primarily use mobile phones for social purposes, while boys prioritize leisure (Lee et al., 2018). This pattern extends to social media use, where poor self-regulation leads 21% of adolescents to addictive behaviors, linked to anxiety, cyberbullying, and substance use (Cimadevilla et al., 2019; Machado et al., 2018). Furthermore, Internet Gaming Disorder, affecting 2.8% of adolescents, is associated with social exclusion, low self-esteem, and executive dysfunction (Warburton et al., 2022). The potential for video game addiction is driven by unmet psychological needs, such as achievement or socialization, and may exacerbate psychotic disorders in vulnerable adolescents (Bekir and Çelik, 2019; Ricci et al., 2023).

Adolescent boys over 16 show a higher prevalence of gambling behaviors (Caselles et al., 2018; Hing et al., 2022). Impulsivity and sensation-seeking often motivate this activity, frequently linked to alcohol and substance use, particularly in online gambling. Cocaine use, while less prevalent, is associated with other risk behaviors like online gambling, with adolescent gamblers twice as likely to use cocaine compared to the general population (Isorna et al., 2022).

The phenomenon of polyconsumption in adolescents highlights the interaction of social, familial, and psychological factors. Motivations include intrinsic ones (e.g., fun, disinhibition) and extrinsic ones (e.g., peer pressure), with gender differences: girls often justify these behaviors for social acceptance, whereas boys link them to displays of status (Dos Santos et al., 2018). Family and social environments are key determinants, with economic availability playing a critical role (Díaz et al., 2018). Polyconsumers exhibit deficits in executive functions and dysfunctional coping styles (Villanueva et al., 2019). Early initiation increases the likelihood of transitioning to stronger substances, such as opioids and cocaine (Jones and McCance-Katz, 2019). Moreover, technological dependency exacerbates alcohol and tobacco consumption (Cimadevilla et al., 2019).

### 3.5 Qualitative analysis of selected studies

**Figure 2:**
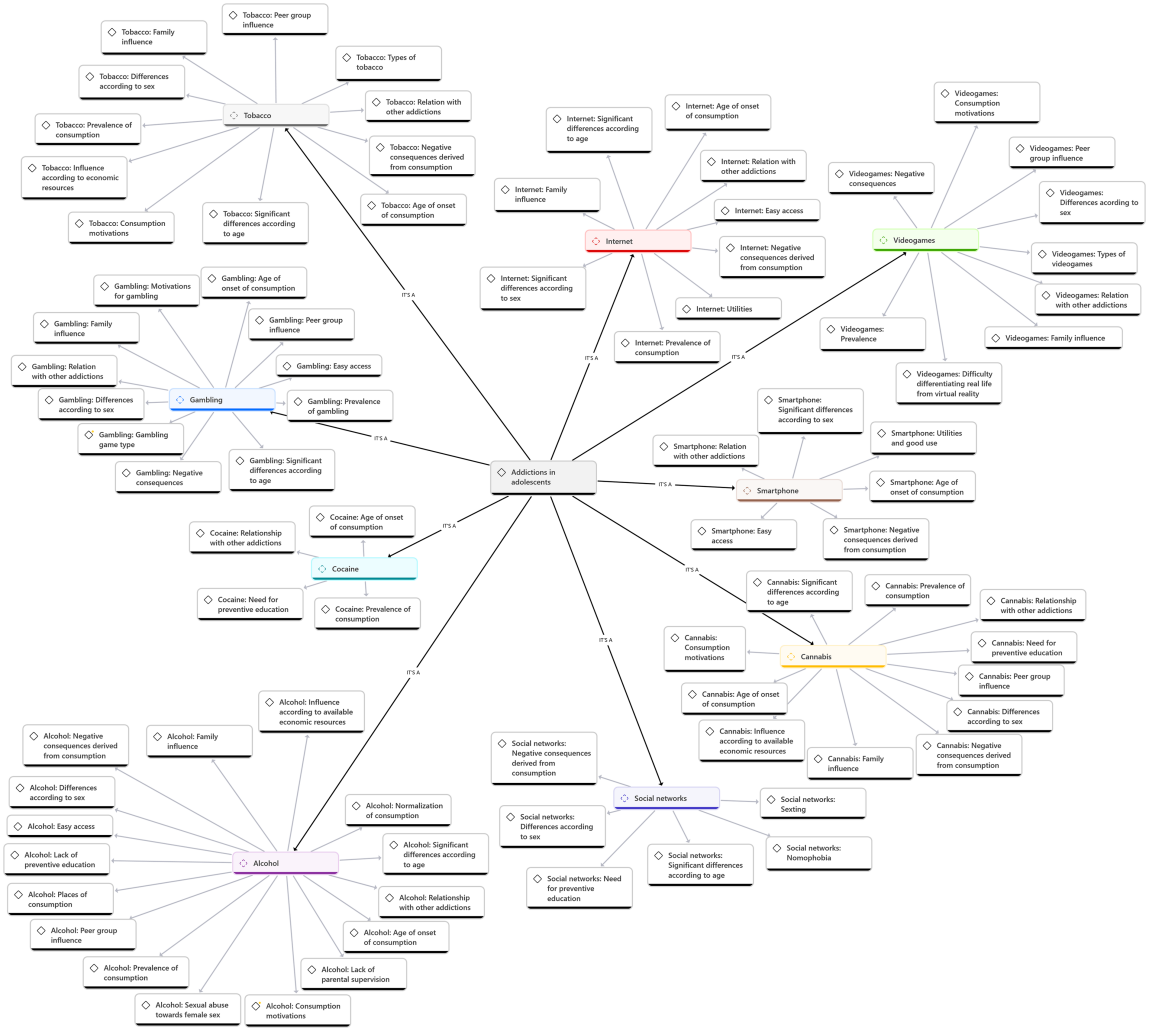
Qualitative analysis of the results obtained.

When analyzing the selected documents, the most studied addictions over the past 5 years were identified as alcohol, tobacco, cannabis, cocaine, video games, gambling, internet, social networks, and smartphones. To achieve the main objective of this systematic review, a qualitative analysis was conducted using the technological tool ATLAS.ti Web (version 3.15.0; ATLAS.ti Scientific Software Development GmbH, 2022). This process provided more rigorous information on the subtopics addressed within each addiction (see Figure 2).

Overall, the results indicate that an early onset of substance use increases the risk of addiction in subsequent years (Hernández et al., 2022; Villanueva et al., 2019). The risk of developing an addiction also increases with age and is more prevalent among males and individuals with greater economic resources (Díaz et al., 2018). The motivations for consumption are influenced by interpersonal and sociocultural factors (Dos Santos et al., 2018).

## 4 Discussion

### 4.1 Substance use

Adolescence is a critical life period characterized by ongoing biological, behavioral, and social transformations that drive human development (Bustamante et al., 2022; Zammit et al., 2018). Changes in brain structure and function, along with influences from the social environment, can increase the likelihood of adolescents engaging in problematic behaviors (Viejo and Ortega-Ruiz, 2015). This makes adolescence a stage particularly prone to risky and reckless behaviors (Steinberg, 2008).

Tobacco and alcohol use represent the most common types of substance abuse among European adolescents (Dudok and Piko, 2023). Motivations for consumption are influenced by personal, social, and cultural factors (Dos Santos et al., 2018; Hernández et al., 2022). The availability and consumption of addictive substances pose a serious social problem, leading to significant health disorders for young people and affecting their environment (Pérez-Fuentes et al., 2018). The initial age of alcohol consumption is around 13.4 years (Rial et al., 2020).

Following this first experience, prevalence increases notably, with consumption often escalating from sporadic to habitual and excessive (Martínez-Fernández et al., 2018; González-Ramos et al., 2018; Rodrigues et al., 2018). Addiction to alcohol results in various negative consequences for minors, primarily impacting their physical, mental, and emotional health, and can even lead to school failure (Calero et al., 2019; Clements-Nolle et al., 2019; González-Ramos et al., 2018; Wartberg et al., 2021; Wicki et al., 2018) and violent situations. Another significant problem associated with alcohol is its normalization in society (Villanueva et al., 2019), the ease of acquisition, and the various places of consumption, which include not only social gatherings but also one’s own home (Rodrigues et al., 2018).

Tobacco dependence among adolescents has been observed to have an earlier onset compared to other substances (Barragán et al., 2016; Villanueva et al., 2019), and shows considerable prevalence (Martínez-Fernández et al., 2018).

An increase in prevalence with age has been observed: according to the results of the HBSC Study (Ministerio de Sanidad, 2018), the percentage of individuals who report daily tobacco use increases with age, peaking among 17-18-year-olds. At this age, minimal differences in consumption are observed between males and females.

Various forms of tobacco use have been identified, including cigarettes, cigars, water pipes, vapes, and e-cigarettes, with the latter being the most prominent according to recent research. The motivation for consumption appears to be driven by sensation seeking (Camelo et al., 2019; Martínez-Fernández et al., 2018; Silveira et al., 2019; Strong et al., 2019; Tackett et al., 2021). Negative impacts include increased impulsivity (González-Roz et al., 2021) and a higher propensity for other risky behaviors (Demissie et al., 2017). The peer relationships and peer pressure significantly influence the acquisition of this addiction (Villanueva et al., 2019), along with academic failure. Family structure also plays a crucial role, particularly in reconstructed families (Camelo et al., 2019; Isorna et al., 2022), where age and gender are significant factors, with older males being more likely to consume (Díaz et al., 2018; Peruga et al., 2022). Finally, economic availability directly influences consumption, as adolescents with greater financial resources tend to consume more (Díaz et al., 2018).

The average age of onset for cannabis use is around 13 years, with the mean age for daily use being approximately 13.8 years (Matalí et al., 2018). Over time, there has been an observed increase in the prevalence of cannabis use (Martínez-Fernández et al., 2018). The motivation for consuming this substance appears to be linked to sensation seeking (Camelo et al., 2019; Martínez-Fernández et al., 2018; Silveira et al., 2019), as well as a desire to escape from situations that generate negative feelings or to achieve a state of relaxation (Matalí et al., 2018). Among the negative consequences of cannabis consumption, low self-esteem (Camelo et al., 2019), increased impulsivity (González-Roz et al., 2021), and the manifestation of violent and problematic behaviors (Villanueva et al., 2019) stand out.

According to the ESPAD report, cannabis is the most consumed illicit substance among European students (Ministerio de Sanidad, 2020). They are considered at risk of severe dependence, a trend potentially driven by the emergence of new variants of cannabis and the increasing potency of its derivatives in recent years (Ministerio de Sanidad, 2020).

Cannabis use by adolescents is influenced by several factors, including family background, belonging to reconstructed families, economic resources, and peer groups. Peer pressure exerts a dual influence: surrounding oneself with friends who consume increases the likelihood of addiction (Hahlbeck and Vito, 2022), while adolescents lacking adequate social skills experience fear and pressure due to the potential rejection by their peers (Matalí et al., 2018; Villanueva et al., 2019).

The results of the HBSC Study emphasize that cannabis use is more prevalent among males than females, particularly within the 17-18-year-old demographic (Ministerio de Sanidad, 2018).

Additionally, age, duration of use, and economic status also negatively impact cannabis use among minors (Díaz et al., 2018). Cannabis use is often associated with the poly-consumption of substances harmful to health (Jones and McCance-Katz, 2019).

Due to the growing concern about addiction to both traditional and new behavioral substances, several studies have analyzed this issue. For instance, Salamó et al. (2010) investigate alcohol consumption patterns, social influence, and risk perception, while Rial et al. (2020) examine the impact of early onset of consumption and identify related variables, such as substance use within the family environment or among peers. Additionally, the consumption of not only traditional cigarettes but also electronic or vapor cigarettes has become prevalent (Demissie et al., 2017).

Other studies have explored polydrug use among adolescents. For example, Barragán et al. (2016) examine the factors associated with alcohol, tobacco, and cannabis use in high school students. Similarly, Peruga et al. (2022) analyze the use of electronic cigarettes and alcohol in high school students, while Calero-Plaza et al. (2020) investigate adolescent poly-consumption of alcohol, cannabis, and marijuana, linking it to violent and delinquent behaviors. In addition, Isorna et al. (2022) analyze addiction to the Internet, gambling, tobacco, alcohol, cocaine, and cannabis.

On the other hand, behavioral addictions are defined as impulsive and repetitive behaviors characterized by a loss of control, a strong compulsive desire to engage in them, persistent emotional discomfort, and continuation of the behavior despite its serious consequences (De Sola et al., 2013).

### 4.2 Problematic use of technology and behavioral addictions

Adolescents use Information and Communication Technologies (ICTs) more frequently and for longer periods of time, often without adequate parental supervision, in various settings such as education, home, or leisure time. They use these tools for a range of purposes, from maintaining social relationships to performing academic tasks or entertaining themselves, either alone or with friends, which can lead to problematic use, especially between the ages of 12 and 17 (Gairín and Mercader, 2018). Consequently, some studies analyze problematic internet use based on risk profiles, family environment characteristics, and behavioral and psychological factors (Arrivillaga et al., 2021). The average age of onset of daily internet use is between 6 and 15 years old (Kaya and Dalgiç, 2021), and the prevalence of internet addiction among adolescents is notable, reaching 21% (Machado et al., 2018). Negative consequences of internet use include low self-esteem (Peris et al., 2018) and lack of self-regulation (Pastor et al., 2022). Significant data related to internet addiction have been observed, such as a higher risk among males at the age of 17, despite an increasing prevalence among females, and low emotional regulation (Arrivillaga et al., 2021). Additionally, the importance of parental control in reducing addictive behaviors is highlighted (Isorna et al., 2022).

Gambling, which involves the use of money or bets, typically begins between the ages of 12 and 13 (Caselles et al., 2018; Isorna et al., 2022). Studies, such as Hing et al. (2022), highlight the ease of access to these games for adolescents. A high prevalence of this activity is observed among adolescents, exacerbated by the normalization of gambling within their immediate environment (Caselles et al., 2018). The primary motivations for gambling include stress management, socialization, and the pursuit of positive emotions (Grande-Gosende et al., 2019). Studies indicate that various types of gambling are readily available and easily accessible (Martínez-Loredo et al., 2019). This addiction is also influenced by the minor’s age, gender, and the social acceptance of gambling within both the family environment and peer groups (Caselles et al., 2018).

Playing video games is not inherently negative, but it can sometimes develop into a pathological practice (Hidalgo-Fuentes, 2023a; Pereira, 2021). The estimated global prevalence of gaming addiction is 3.5% (Ricci et al., 2023), and studies such as Buja et al. (2020) estimate that nearly a third of gamers experience difficulties related to playing video games. The motivations behind this problem include fun, the search for new sensations, the ease of playing from home with peers, the constant positive reinforcement from prizes and winning, and the adventure experience it provides, fulfilling the psychological needs of adolescents (Bekir and Çelik, 2019). Video games help them manage stress and develop positive emotions (Grande-Gosende et al., 2019), serving as a method of coping with social difficulties (López-Fernández et al., 2020). Male adolescents, particularly younger ones, are most associated with this problematic practice (Macur and Pontes, 2021; Warburton et al., 2022). Factors such as executive dysfunction, lack of self-regulation, impulsivity, and unmet needs outside the virtual world contribute to this issue. Adolescents from disconnected and disaffected family environments are also at increased risk (López-Fernández et al., 2020; Warburton et al., 2022). Other studies highlight profiles with low agreeableness and responsibility in males, and low extraversion and responsibility in females (López-Fernández et al., 2021). The repercussions of excessive video game playing include decreased self-control (Macur and Pontes, 2021), altered sleep patterns, and an increased risk of engaging in harmful behaviors such as substance use, alcohol, tobacco, or gambling on slot machines (Cimadevilla et al., 2019). Additionally, a correlation has been observed between excessive video game playing and mental health symptoms, as well as increased severity in behavioral problems (Richard et al., 2023).

Adolescents at high risk of smartphone addiction exhibited significantly more severe levels of psychopathologies across all dimensions of the Youth Self-Report scale (Lee et al., 2018). This addiction affects psychological factors such as anxiety, behavioral inhibition system, and aggression (Lee et al., 2020), leading to a greater tendency toward risky behaviors and lower self-control, particularly in girls (Dou et al., 2020).

Lastly, social networks have a significant influence on daily life and are widely used by adolescents. Some individuals are more susceptible to developing addictions to these platforms, particularly those with an introverted profile (Muñoz and Ramírez, 2016). It has been observed that females are more likely to experience symptoms of addiction, excessive use, and nomophobia compared to males, with these tendencies being more pronounced in the age range of 12 to 14 years (Peris et al., 2018). This phenomenon can adversely impact the mental health of adolescents, as it encourages constant comparison with others, decreases self-esteem, increases exposure to online bullying, and promotes dependence and social isolation, all of which affect emotional well-being (Herrera-Peco et al., 2023).

This study synthesizes findings from a range of sources to provide a comprehensive and multidimensional perspective on adolescent substance use initiation and abuse. By analyzing substance use alongside behavioral patterns, it identifies emerging trends and offers actionable insights to inform the development of evidence-based prevention and intervention strategies. The findings emphasize the need for tailored prevention approaches that address early onset, gender-specific risk factors, and the influence of family environment and peer relationships.

For clinicians, the study underscores the importance of incorporating routine screenings for substance use and behavioral addictions into adolescent health assessments. For educators and policymakers, it provides critical insights to design targeted educational campaigns and enforce policies that limit access to harmful substances. This integrative approach fills significant gaps in current research, offering valuable guidance to stakeholders aiming to reduce the risks associated with adolescent substance use and behavioral addictions.

## 5 Conclusion

This systematic review has identified research addressing the most common substance abuse and digital addictions among adolescents, providing a comprehensive analysis of this issue. This review has successfully fulfilled its primary objective of analyzing updated information to describe the most prevalent substance use and behavioral patterns among adolescents. By examining the most relevant data on this problem, it serves as a foundation for prevention and intervention projects aimed at reducing or eliminating these behaviors, which are pervasive in the daily lives of young people and detrimental to their physical and psychological health.

This study has presented several limitations. Despite the large number of studies found, most methodologies used were quantitative in nature. This limits a more descriptive research development that could address more individualized cases. However, the quantitative nature of these studies has allowed for the representation of results from a larger sample of students.

Additionally, this review may be affected by potential publication bias, the limited number of longitudinal studies, and the exclusion of research not available in the databases consulted. These factors could influence the comprehensiveness of the findings and highlight the need for more diverse and longitudinal research in the future.

To address substance abuse and digital addictions in adolescents, it is vital to implement comprehensive education that encompasses biological, psychological, social, and cultural aspects. This includes promoting coping skills, critical thinking, and resistance to group influence, as well as utilizing educational technology and fostering interdisciplinary collaboration. In light of the strong influence that both positive and negative peer relationships exert on adolescent behavior, future research and interventions should explore the potential of peer-based programs. Such initiatives—whether in school, community, or online settings—can harness peer support to promote healthy behaviors and reduce the impact of peer pressure. Evidence from health promotion and prevention studies suggests that well-structured peer-led approaches can enhance self-efficacy, foster positive social norms, and serve as an effective complement to family- and school-based strategies. Therefore, it is essential to develop prevention and intervention programs to curb this problem, and to provide minors and their immediate environment with sufficient information about the risks and consequences of both substance use and behavioral addictions.

## Data Availability

The original contributions presented in the study are included in the article/supplementary material, further inquiries can be directed to the corresponding author.
